# Dataset of non-pharmaceutical interventions and community support measures across Canadian universities and colleges during COVID-19 in 2020

**DOI:** 10.3389/fpubh.2022.1066654

**Published:** 2022-11-17

**Authors:** Haleema Ahmed, Taylor Cargill, Nicola Luigi Bragazzi, Jude Dzevela Kong

**Affiliations:** ^1^Kong Research Group, Department of Biology, Faculty of Science, York University, Toronto, ON, Canada; ^2^Africa-Canada Artificial Intelligence and Data Innovation Consortium, York University, Toronto, ON, Canada; ^3^Laboratory for Industrial and Applied Mathematics, Department of Mathematics and Statistics, Faculty of Science, York University, Toronto, ON, Canada; ^4^Kong Research Group, Department of Mathematics and Statistics, Faculty of Science, York University, Toronto, ON, Canada

**Keywords:** COVID-19, pandemic, policy, post-secondary, university, dataset, Canada, non-pharmaceutical intervention

## Introduction

In Canada, the first confirmed case of “Coronavirus Disease 2019” (COVID-19), the disease caused by the virus known as “Severe Acute Respiratory Syndrome-related Coronavirus type 2” (SARS-CoV-2), was reported on January 25, 2020. COVID-19 was declared a Public Health Emergency of International Concern (PHEIC) by the World Health Organization (WHO) only 5 days later, on January 30th, and later a global pandemic on March 11, 2020 (1). Without widespread availability of effective COVID-19 vaccines or treatments in Canada, the government relied on non-pharmaceutical intervention (NPI) measures as the primary mitigation strategy for slowing the spread of COVID-19 (2). Canadian post-secondary institutions were faced with the challenge of interpreting the NPI guidance and announcements issued from federal, provincial and local public health authorities as well as decision and policy makers. However, guidance in some regions was regularly revised and/or updated rapidly to reflect the constantly evolving nature of the COVID-19 situation and the gradual accumulation of information on COVID-19 virulence and transmission. Thus, schools were, to some degree, called upon to take an individualized, proactive approach in deciding which NPI decisions to implement and when to implement them (3). In order to address the unique situation of their campus and community, institutions layered multiple COVID-19 mitigation strategies based on what each school deemed necessary for a robust institution-wide response. This process was typically directed by committees composed of university/college leadership, and it involved careful balancing of economic concerns, recommendations by public health authorities, and the needs of students, faculty and staff.

The majority of institutions communicated NPI decisions regularly to their internal student-staff community as well as the wider public through institution websites and social media channels. However, information on the reasoning and context behind these decisions is less typically made public. While studies have been conducted on factors affecting NPI adoption timing for universities in the United States of America, similar research has not been conducted in the context of Canada. Compiling the first dataset on the status and timing of NPI decisions and community support measures made by post-secondary institutions in response to the COVID-19 pandemic is valuable in illuminating for future study, why institutions made certain decisions, how effective these decisions were in containing viral spread, whether these decisions were data-driven and locally-informed, and how these choices intersected with the broader Canadian political and socio-economic landscape of COVID-19. With this aim, this study provides a dataset on the timing of 17 NPI decisions and support measures made by 122 post-secondary institutions throughout the year 2020.

## Methods

An original database was manually compiled for 122 of 382 recognized universities and colleges in Canada (4). This includes the representation of universities and colleges from 10 provinces and 2 territories. The authors used a stringent list of inclusion/exclusion criteria for defining decision categories. This criteria legend is appended to the database.

This study was modeled from another study conducted by researchers at George Mason University (GMU) on 5 NPI observations in 575 universities during March 2020 (5). GMU researchers similarly searched institutional web pages selected from the Department of Education Integrated Postsecondary Education Data System in the U.S. This study elaborated on their methodology extending the number of NPI variables and including community support measures to obtain a greater insight on the specific pandemic-responses of post-secondary institutions. Rather than capturing policy announcements from March 2020 only, this study was also extended to the entire 2020 year to provide a high-level capture of the first year of COVID-19 with more NPI variables and community support measures that became relevant during the later months of the pandemic. Additionally, this extension allowed collection of data during the initial pandemic panic stage and as society moved into the “new normal” stage.

The institutions selected for this study were first selected from both Eduvation and University Affairs. Eduvation, a Canadian organization that tracks trends in higher education, provides a publicly available database for COVID-19 campus data including vaccine requirements for major institutions (6). University Affairs provides news and commentary on Canadian universities including COVID-19 updates (7). In both comparing and combining the institutions listed by these two major sources of post-secondary information in Canada, a comprehensive selection of universities and colleges was developed.

The variable was first marked with 1 (YES) or 0 (NO) to signify whether an affirmative decision regarding that variable was announced during the year 2020. If an affirmative decision was made, the date of the announcement (and for some variables, the effective date of the decision) was included in the database. A dash (-) was used for data points that did not have information available even after contacting the institution, and an NA (not applicable) was also utilized when necessary.

## Data acquisition

Many institutions provided a COVID-19 webpage with chronological policy updates for their students, faculty, employees, and the wider community who utilize their services. Other institutions published designated COVID-19 updates on their institution news page which included announcements unrelated to the pandemic. These sources were first utilized when searching for the relevant data. In the case where NPI announcement data was not available on institutional COVID-19 websites or news pages, social media channels were combed through and the institution was contacted by email for further information and to complete any gaps.

## Preliminary analysis

A total of 122 post-secondary institutions, 13 NPI variables and 4 community support measures were included in this preliminary descriptive analysis after quality control. The most robustly-captured decisions were changes in convocation planning (98.4% of school decisions captured), moving online (97.5%), mental health support (94.3%) and Fall 2020 mostly remote (89.3%). The remaining NPI variables and community support measures were captured in decision announcements for 66.4 to 88.5% of schools in the dataset. The least captured decisions were cancellation of international travel (68.9%), technology access (67.2%), discouragement of on-campus housing (67.2%) and PPE distribution (66.4%). In cases where publicly-available information on the timing of the decision could not be found, some variables have a proportion of school “YES” decisions that lack corresponding dates. Table 1 describes the summary statistics for each variable in the dataset.

**Table 1:** Mean announcement dates and percentage (%) of Canadian post-secondary schools with “Yes” decisions to non-pharmaceutical interventions (NPI) and community support measures in 2020.

**Province (# schools)**	**Remotework**		**Campusclosure**		**Moveonline**		**Class suspended**		**Discourage housing**		**Canceltravel**		**PPEenforcement**		**PPEdistribution**	
	**%**	**Mean**	**%**	**Mean**	**%**	**Mean**	**%**	**Mean**	**%**	**Mean**	**%**	**Mean**	**%**	**Mean**	**%**	**Mean**
AB (18)	78	Mar 16	67	Apr 4	89	Mar 14	89	Mar 14	50	Mar 19	56	Mar 7	78	Aug 5	50	Aug 12
BC (21)	86	Mar 18	90	Mar 23	100	Mar 15	57	Mar 15	48	Mar 23	71	Mar 12	95	Oct 3	62	Aug 28
MB (7)	100	Mar 22	100	Mar 23	100	Mar 14	86	Mar 13	43	Mar 15	71	Mar 14	100	Aug 16	100	Aug 13
NB (4)	100	Mar 18	100	Mar 16	100	Mar 13	100	Mar 13	100	Mar 16	100	Mar 22	75	Sep 6	50	Aug 31
NL (2)	50	Mar 17	100	Mar 31	100	Mar 15	100	Mar 15	100	Mar 15	100	Mar 8	100	Sep 21	50	Aug 21
NWT (1)	100	Mar 20	100	Mar 21	100	Mar 18	100	Mar 18	100	Mar 17	0	NA	0	NA	0	NA
NS (9)	100	Mar 18	78	Mar 17	100	Mar 14	100	Mar 14	78	Mar 14	78	Mar 13	78	Aug 11	67	Jul 23
ON (46)	89	Mar 15	91	Mar 18	100	Mar 13	91	Mar 13	78	Mar 16	67	Mar 13	87	Jul 29	67	Jul 10
PE (2)	100	Mar 17	100	Mar 16	100	Mar 15	100	Mar 15	100	Mar 16	100	Mar 15	100	Jun 17	100	Jun 17
QC (5)	100	Mar 13	100	Mar 13	100	Mar 14	100	Mar 12	100	Mar 21	100	Mar 14	100	Jul 14	80	Aug 30
SK (6)	83	Mar 18	83	Mar 19	100	Mar 14	100	Mar 14	50	Mar 16	50	Mar 16	100	Aug 16	83	Aug 17
YT (1)	100	Mar 16	100	Mar 17	100	Mar 16	0	NA	0	NA	0	NA	100	Aug 17	100	Nov 23
**Province (# schools)**	**Limited library access**		**Technologyaccess**		**Increased sanitation protocol**		**Mental health support**		**Financial support**		**Convocation change**		**Fall 2020 mostly remote**		**Winter 2021 mostly remote**	
	**%**	**Mean**	**%**	**Mean**	**%**	**Mean**	**%**	**Mean**	**%**	**Mean**	**%**	**Mean**	**%**	**Mean**	**%**	**Mean**
AB (18)	78	Mar 18	61	Apr 7	67	Mar 23	100	Mar 24	67	Apr 30	100	Apr 4	89	Jun 2	72	Sep 28
BC (21)	100	Mar 19	76	Apr 20	86	Apr 11	95	Mar 20	90	Apr 5	95	Apr 1	100	May 21	90	Sep 19
MB (7)	86	Mar 18	71	Apr 20	100	Mar 13	71	Apr 3	57	Apr 4	100	Mar 27	86	May 28	86	Oct 4
NB (4)	100	Mar 18	25	Mar 16	75	Mar 14	100	Mar 5	75	Mar 31	100	Mar 20	75	Jun 15	75	Oct 4
NL (2)	50	Mar 17	100	Jun 1	50	Mar 16	100	Mar 26	100	Apr 18	100	Mar 23	50	May 10	50	Oct 10
NWT (1)	100	Mar 20	100	Oct 8	0	NA	100	Mar 24	0	NA	100	Mar 21	100	Jun 16	100	Oct 8
NS (9)	56	Mar 16	44	Mar 23	89	Mar 31	89	Mar 7	100	Apr 4	100	Mar 16	78	May 22	56	Sep 22
ON (46)	85	Mar 16	72	Apr 10	83	Mar 25	96	Mar 21	93	Apr 10	98	Mar 31	96	Jun 1	89	Oct 4
PE (2)	100	Mar 17	100	Mar 16	100	Apr 20	100	Feb 23	100	Apr 25	100	Mar 31	100	May 20	50	Sep 25
QC (5)	80	Mar 14	40	Mar 22	100	Apr 15	100	Apr 6	100	Mar 30	100	Apr 11	60	May 12	80	Oct 19
SK (6)	83	Mar 17	67	Mar 17	100	May 15	83	Mar 22	83	Apr 21	100	Mar 20	67	May 16	83	Sep 10
YT (1)	100	Mar 17	100	Aug 3	0	NA	100	Oct 6	100	May 14	100	Mar 27	100	May 26	0	NA

**Figure 1:**
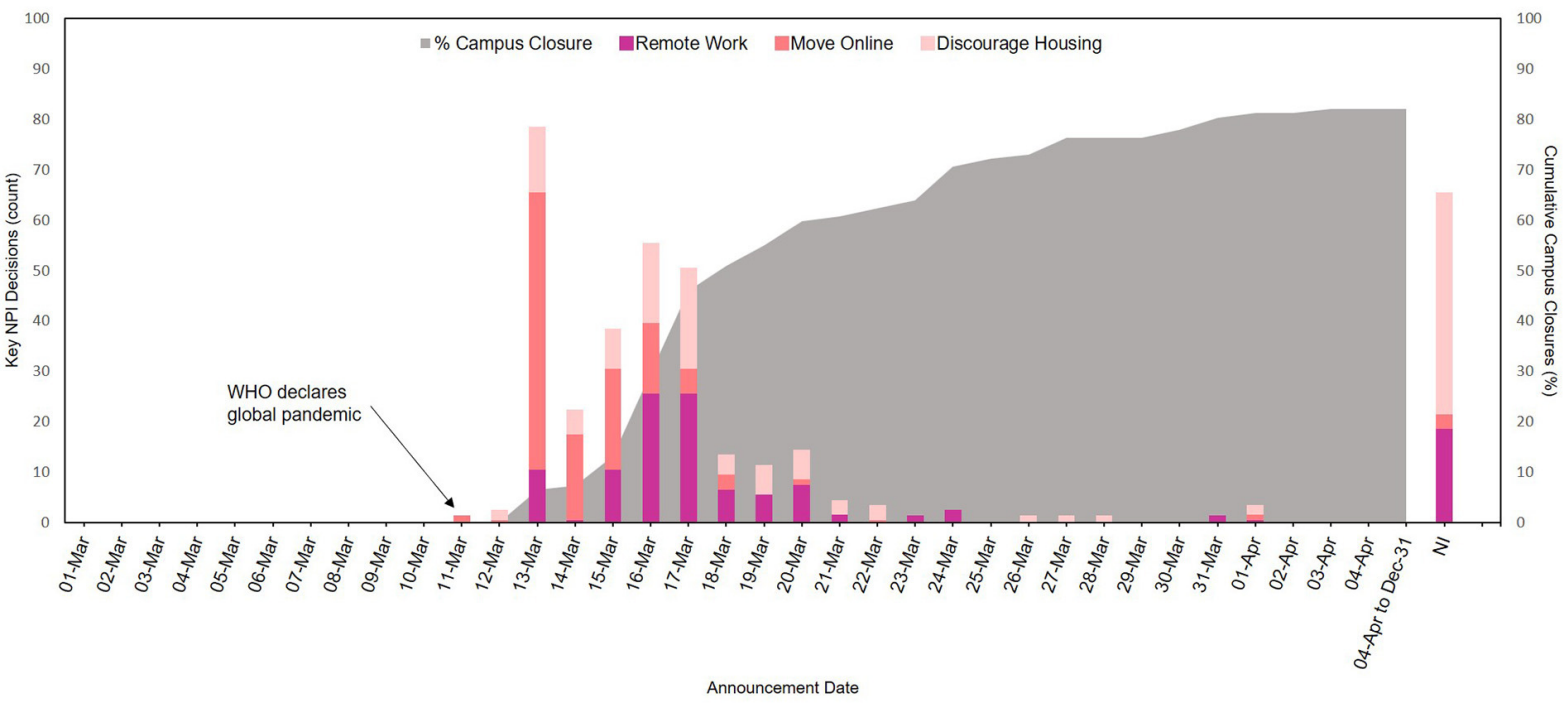
Timeline of four key non-pharmaceutical intervention (NPI) decisions made by Canadian post-secondary institutions in response to the COVID-19 pandemic in 2020.

Figure 1 depicts the timing of Canadian post-secondary schools in making key NPI decisions regarding campus closure to essential personnel, transitioning faculty and staff to remote work, moving classes online, and discouraging on-campus housing, during the year 2020. For the 119 schools that had announcements available concerning their decision of whether to transition to online learning, all moved online between March 11, 2020 and April 1, 2020. The majority of decisions to move classes online occurred on March 13^th^, 2 days after the World Health Organization (WHO) declared COVID-19 a global pandemic. Among the schools for which dated remote work announcements could be obtained (88.5%), all implemented this mitigation strategy by March 31st. Schools began closing their campuses to non-essential personnel by March 13, 2020, and 50% of schools announced campus closure by March 18, 2020.

Six (6) key “shutdown” decisions made by post-secondary schools were: remote work, non-essential campus closure, moving online, suspension of classes, discouragement of on-campus housing, and cancellation of international travel. By province, ≥50% of schools made at least one “shutdown” decision by March 8, 2020 in NL; by March 12 in QB; by March 13 in BC, AB, MB, ON, NB and NS; by March 14 in PEI and SK; by March 16 in YK; and by March 17 in the NWT. For the four “community support” decisions (PPE distribution, technology access, mental health support and financial support), by province, ≥50% of schools made at least one of these “community support” decisions by February 23, 2020 in PEI; by March 16 in NS; by March 17 in ON and NB; by March 18 in AB, QB and SK; by March 20 in BC and MB; by March 24 in the NWT; by March 26 in NL; and by May 14 in YK.

Of the support measures considered by this study, mental health supports were the most highly implemented, with 94.3% of Canadian post-secondary schools making an announcement to provide mental health resources to their campus community. In total of 86.1% of schools provided students with internally-offered financial support, such as a COVID-19 Emergency Bursary. Roughly 66–67% of schools announced measures to support students/faculty with overcoming technology access barriers and accessing masks or face coverings.

## Implications

While Canadian public health operates across national, provincial, and local levels, each province or territory has oversight over their healthcare systems thus allowing specific mitigation strategies to operate. This research provides opportunities for provincial and territorial comparisons on the rates and mean announcement dates of NPIs and community support measures. As the federal government handles pandemic related decisions such as travel and financial support, variability across and within provinces and territories can be attributed to provincial and regional public health authority dialogue. Despite the relatively civil cooperation across Canadian public health authorities, criticism from experts and specialists on decisions invariably existed resulting in layers of decision making (8).

In many cases, post-secondary school decisions appear to have been highly informed by the recommendations which were regularly made by their respective provincial public health units. For instance, during data collection, it was noted that some announcements classified as “PPE Enforcement” and “Fall 2020 Mostly Remote” tended to directly reference most recent guidance from provincial public health authorities. Thus, the province in which a school was situated may have had varying degrees of influence on the timing of NPI and community support decisions made by schools. Further investigation into the influence of external factors such as province and other location-based characteristics on post-secondary decisions may lead to insights into the extent to which schools relied on external guidance for their decision-making.

While mental health support decisions were announced by 94.3% of institutions across Canada, the mean dates of these announcements varied widely across the country (Table 1). Further analysis of the dataset may inform the factors influencing this variance and whether the mental health supports provided by institutions were effective.

Further analysis on the dataset produced for this study can shed light into a much-needed discussion on the Canadian COVID-19 pandemic response in education.

## Data availability statement

The original contributions presented in the study are included in the article/Supplementary material, further inquiries can be directed to the corresponding author/s.

## Author contributions

TC and HA conducted the literature research, compiled the dataset, conducted the data analysis, and drafted the manuscript. NLB and JDK designed and supervised the study. All authors approved the final manuscript.

## Funding

JDK and NLB acknowledge support from IDRC (Grant No. 109981). JDK acknowledges support from New Frontier in Research Fund Exploratory (Grant No. NFRFE-2021-00879) and NSERC discovery grant (Grant No. RGPIN-2022-04559). HA acknowledges support from the York University Science Scholars Award. TC acknowledges support from the Research at York program and The Bahamas National Merit Scholarship.

## Conflict of interest

The authors declare that the research was conducted in the absence of any commercial or financial relationships that could be construed as a potential conflict of interest.

## Publisher's note

All claims expressed in this article are solely those of the authors and do not necessarily represent those of their affiliated organizations, or those of the publisher, the editors and the reviewers. Any product that may be evaluated in this article, or claim that may be made by its manufacturer, is not guaranteed or endorsed by the publisher.
